# Ligands Exert Biased Activity to Regulate Sigma 1 Receptor Interactions With Cationic TRPA1, TRPV1, and TRPM8 Channels

**DOI:** 10.3389/fphar.2019.00634

**Published:** 2019-06-12

**Authors:** Elsa Cortés-Montero, Pilar Sánchez-Blázquez, Yara Onetti, Manuel Merlos, Javier Garzón

**Affiliations:** ^1^Neuropharmacology, Department of Translational Neuroscience, Cajal Institute, CSIC, Madrid, Spain; ^2^Drug Discovery & Preclinical Development, Esteve, Barcelona, Spain

**Keywords:** type 1 sigma receptor, transient receptor potential ankyrin member 1, transient receptor potential melastatin member 8, transient receptor potential vanilloid member 1, calmodulin, histidine triad nucleotide-binding protein 1, ligand bias, *N*-methyl-D-aspartate receptor

## Abstract

The sigma 1 receptor (σ1R) and the mu-opioid receptor (MOR) regulate the transient receptor potential (TRP) V1 calcium channel. A series of proteins are involved in the cross-regulation between MORs and calcium channels like the glutamate *N*-methyl-D-aspartate receptor (NMDAR), including the histidine triad nucleotide-binding protein 1 (HINT1), calmodulin (CaM), and the σ1R. Thus, we assessed whether similar mechanisms also apply to the neural TRP ankyrin member 1 (TRPA1), TRP vanilloid member 1 (TRPV1), and TRP melastatin member 8 (TRPM8). Our results indicate that σ1R and CaM bound directly to cytosolic regions of these TRPs, and this binding increased in the presence of calcium. By contrast, the association of HINT1 with these TRPs was moderately dependent on calcium. The σ1R always competed with CaM for binding to the TRPs, except for its binding to the TRPA1 C-terminal where σ1R binding cooperated with that of CaM. However, σ1R dampened HINT1 binding to the TRPA1 N-terminal. When the effect of σ1R ligands was addressed, the σ1R agonists PRE084 and pregnenolone sulfate enhanced the association of the σ1R with the TRPM8 N-terminal and TRPV1 C-terminal in the presence of physiological calcium, as seen for the σ1R–NMDAR interactions. However, these agonists dampened σ1R binding to the TRPA1 and TRPV1 N-terminal domains, and also to the TRPA1 C-terminal, as seen for σ1R–binding immunoglobulin protein (BiP) interactions in the endoplasmic reticulum (ER). By contrast, the σ1R antagonists progesterone and S1RA reduced the association of σ1R with TRPA1 and TRPV1 C-terminal regions, as seen for the σ1R–NMDAR interactions. Conversely, they enhanced the σ1R interaction with the TRPA1 N-terminal, as seen for σ1R–BiP interactions, whereas they barely affected the association of σ1R with the TRPV1 N-terminal. Thus, depending on the calcium channel and the cytosolic region examined, the σ1R agonists pregnenolone sulfate and PRE084 opposed or collaborated with the σ1R antagonists progesterone and S1RA to disrupt or promote such interactions. Through the use of cloned cytosolic regions of selected TRP calcium channels, we were able to demonstrate that σ1R ligands exhibit biased activity to regulate particular σ1R interactions with other proteins. Since σ1Rs are implicated in essential physiological processes, exploiting such ligand biases may represent a means to develop more selective and efficacious pharmacological interventions.

## Introduction

The sigma 1 receptor (σ1R) is a 223-amino-acid polypeptide that is widely distributed in different tissues and cell compartments. In nervous tissue, the σ1R is located in areas implicated in nociception and pain control, such as the spinal cord ganglia, substantia gelatinosa of the dorsal horn, and brainstem (Kitaichi et al., 2000; Su et al., 2010). Initially, the σ1, mu, and kappa receptors in the neural plasma membrane were pharmacologically classified as opioid receptors (Martin et al., 1976). However, the absence of a G-protein-coupled receptor (GPCR) structure and regulated transduction distanced the σ1R from the opioid receptor family (Su et al., 1988; Su et al., 2010). Nevertheless, the σ1R maintains a relationship with the opioid system, where it exerts a tonic anti-opioid effect (Mei and Pasternak, 2002) and modulates the activity-induced sensitization of nociceptive pathways (Cobos et al., 2008; Maurice and Su, 2009; Diaz et al., 2009). Thus, certain σ1R ligands enhance the antinociceptive effects of clinically relevant mu-opioid receptor (MOR) opioids such as morphine, fentanyl, oxycodone, codeine, buprenorphine, and tramadol (Mei and Pasternak, 2002; Diaz et al., 2009; Sánchez-Fernández et al., 2014). However, although other σ1R ligands do not alter opioid-induced analgesia, they dampen antagonist-mediated effects. Thus, the σ1R ligands that enhance MOR analgesia are referred to as antagonists, and those that reduce opioid analgesia and/or oppose the effects of antagonists are classified as agonists.

Under normal conditions, σ1R antagonists do not alter mechanical or thermal thresholds but instead decrease the perception of pain caused by nociceptive sensitization or by pathological states, such as neuropathy, inflammation, or ischemic pain (Kim et al., 2006; Roh et al., 2008; Romero et al., 2012). Recent research has revealed the presence of σ1Rs in the MOR environment. The cytosolic C-terminus of MOR binds to the histidine triad nucleotide-binding protein 1 (HINT1) protein, facilitating the interactions of the σ1R and glutamate *N*-methyl-D-aspartate receptor (NMDAR) with the MOR (Rodríguez-Muñoz et al., 2015a). In this context, the σ1R cooperates with the HINT1 protein to bring the NMDAR under control of the MOR (Rodríguez-Muñoz et al., 2015b). Indeed, activation of the MOR promotes calcium permeation through the NMDAR, which is regulated by the competitive binding of the σ1R and calmodulin (CaM) to the regulatory cytosolic C1 region of the NMDAR NR1 subunit. Upon σ1R depletion, HINT1 also reduces the inhibitory binding of calcium-activated CaM to the NMDAR NR1 subunit. Therefore, the MOR activates and regulates the function of ionotropic NMDAR calcium channels through the interactions between σ1R, HINT1, and CaM where the calcium-dependent binding of σ1Rs to NMDARs can predominate over the interactions with CaM and HINT1. This observation prompted us to investigate whether other calcium channels may also be regulated by these σ1R-mediated mechanisms.

Different classes of channels in the ER and plasma membrane dynamically control intracellular calcium levels. In the present study, we focus on the transient receptor potential (TRP) channel family, homotetrameric calcium channels with variable cytosolic N- and C-terminal regions that contain diverse regulatory protein binding domains and motifs (Owsianik et al., 2006). CaM binds to the cytosolic N- and C-terminal regions of TRPV1 in a calcium-dependent manner (Numazaki et al., 2003; Rosenbaum et al., 2004), as well as to the C-terminus of TRPA1 (Hasan et al., 2017). In fact, by modulating the gating of the calcium influx, CaM participates in the mechanism regulating TRP activity (Numazaki et al., 2003; Lishko et al., 2007; Sarria et al., 2011; Hasan et al., 2017). In addition, pharmacological interventions targeting σ1R alter TRPV1 expression, with σ1R antagonists downregulating TRPV1 channels in the plasma membrane of sensory neurons (Ortiz-Renteria et al., 2018). Moreover, MOR and TRPV1 channels are co-precipitated when exogenously expressed in cultured cells (Scherer et al., 2017). While nerve damage provoked by peripheral inflammation enhances TRPA1 levels in dorsal root ganglia (DRG) neurons (Obata et al., 2005), TRPV1 levels increase in undamaged sensory connections (Hudson et al., 2001), facilitating the transmission of nociceptive information and thereby contributing to the resulting pain response. Thus, similar to NMDARs, TRP channels play roles in several pain-related pathological conditions, including inflammatory, neuropathic, visceral, and dental pain, as well as in pain associated with cancer (Patapoutian et al., 2009; Julius, 2013; Mickle et al., 2015). Evidence for these roles has mainly been obtained using specific antagonists of individual nociceptive TRP channels in animal models of pain-related pathologies, such as by inducing these pathologies in mice through the genetic deletion/alteration of individual nociceptive TRP channels (Caterina et al., 2000; Davis et al., 2000; Katsura et al., 2006). These studies have led to the development of a new generation of analgesics that target the TRP sensors for heat, cold, and irritants (Kaneko and Szallasi, 2014).

Three TRPs belonging to different subfamilies and expressed at the spinal level and in the brain fulfilled our criteria to be included in a comparative study with the neural NMDAR. The TRP ankyrin member 1 (TRPA1), TRP vanilloid member 1 (TRPV1), and TRP melastatin member 8 (TRPM8) belong to the so-called thermo TRP channels that participate in detecting temperature changes and integrating different noxious stimuli (Julius, 2013). TRPA1 is a non-selective calcium channel activated by multiple stimuli, including harmful cold temperatures, acids, and numerous chemical pollutants (Jordt et al., 2004). The TRPM8 channel plays a physiological role in detecting low temperature (10–33°C), and it is over-expressed in sensory neurons after nerve injury or inflammation; TRPM8 also participates in cold allodynia and hyperalgesia (Xing et al., 2007). TRPV1 is also a non-selective calcium channel that is activated by noxious temperatures (>43°C), an acidic pH, and vanilloid compounds. TRPV1 expression is upregulated in response to acute inflammation (Camprubi-Robles et al., 2009) and in conditions of chronic pain, and the activity of this TRP is potentiated by pro-algetic mediators released during inflammation and tissue injury (Huang et al., 2006). In addition, TRPA1 receptors are coexpressed with TRPV1 channels in C-fiber sensory neurons (Fajardo et al., 2008), and they seem to fulfill crucial roles in neuronal and nonneuronal neuropathic pain.

Accordingly, we addressed whether the cloned N- and C-terminal cytosolic regions of these TRP channels participate in direct and calcium-dependent interactions with the σ1R and the MOR-related HINT1 protein. Because calcium-dependent binding of CaM to cytosolic regions of these TRPs has previously been mapped, we addressed its possible interference in the interaction with σ1Rs. Given the differences that ligands exhibit on the interactions of σ1Rs with BiP in the ER and with NR1 subunits of the NMDAR, we also analyzed their profiles in the interactions of σ1Rs with the cytosolic regions of the TRPs selected. We observed that σ1R interacts with the N- or C-terminus of these TRPs in a calcium-dependent manner, and most relevantly, σ1R ligands exhibit a biased activity to disrupt or promote the interaction of σ1Rs with the TRP domains.

## Materials and Methods

### Recombinant Protein Expression

The coding region of the full-length murine σ1R (AF004927), HINT1 (NM_008248), and the N- and C-terminal regions of TRPA1 (NP_808449; residues 1–721 and 961–1125), TRPV1 (NP_542437; residues 1–433 and 680–839), and TRPM8 (NP_599013; residues 1–639) were amplified by reverse transcription polymerase chain reaction (RT-PCR) using total RNA isolated from the mouse brain as the template. Specific primers containing an upstream Sgf I restriction site and a downstream Pme I restriction site were used, as described previously (Rodríguez-Muñoz et al., 2015b). The PCR products were cloned downstream of the glutathione S-transferase (GST)/HaloTag^®^ coding sequence (Flexi^®^ Vector, Promega, Spain) and the tobacco etch virus protease (TEV) protease site, and when sequenced, the proteins were identical to the GenBank™ sequences. The vector was introduced into the *Escherichia coli* BL21 (KRX #L3002, Promega), and clones were selected on solid medium containing ampicillin. After 3 h of induction at room temperature (RT), in the presence of 1 mM isopropyl β-D-1-thiogalactopyranoside (IPTG) and 0.1% Rhamnose, the cells were collected by centrifugation and maintained at −80°C. The fusion proteins were purified under native conditions on GStrap FF columns (#17-5130-01, GE Healthcare, Spain) or with HaloLink Resin (#G1915, Promega). When necessary, the fusion proteins retained were cleaved on the column with ProTEV protease (#V605A, Promega) and further purification was achieved by high-resolution ion exchange (#780-0001Enrich Q, BioRad, Spain). Sequences were confirmed by automated capillary sequencing. Recombinant calmodulin (CaM, #208694) was purchased at Merck-Millipore (Spain).

### *In Vitro* Interactions Between Recombinant Proteins: Pull-Down of Recombinant Proteins and the Effect of Drugs on the Sigma 1 Receptor– Transient Receptor Potential Interactions

Having demonstrated that the σ1R and HINT1 do not bind to GST (#Z02039; GenScript Co., USA) (see **Supplementary Figure 1**) (Sánchez-Blázquez et al., 2012), we assessed the association of GST-free σ1Rs or HINT1 with the GST-tagged TRP cytosolic sequences. The N- and C-terminal domains of TRP were immobilized through covalent attachment to N-Hydroxysuccinimide (NHS)-activated Sepharose 4 fast flow (4FF, #17-0906-01; GE) according to the manufacturer’s instructions. Recombinant σ1R (200 nM) was then incubated with either NHS-blocked Sepharose 4FF (negative control) or with the immobilized TRP sequence (100 nM) in 200 µL of a buffer containing 50 mM Tris–HCl (pH 7.5) and 0.2% 3-[(3-cholamidopropyl)dimethylammonio]-1-propanesulfonate (CHAPS), and in the presence or absence of 3 mM CaCl_2_. In pilot assays, we found that the TRP–σ1R association was maximal after 30 min incubation and that, in this period, the drugs could also promote stable changes in this association. The samples were mixed by rotation for 30 min at RT, and the σ1Rs bound to TRP-Sepharose 4FF were recovered by centrifugation and washed three times. This protocol was also carried out to assess the TRP-HINT1 or TRP-CaM associations, or the competition between HINT1/CaM and higher concentrations of σ1R to bind to the TRPs. To study whether the drugs used provoked changes in the TRP–σ1R association, the agarose-attached TRP–σ1R complexes were incubated for a further 30 min at RT with rotation in the presence of increasing concentrations of the drugs and in a final reaction volume of 300 µL of 50 mM Tris–HCl (pH 7.5), 3 mM CaCl_2_ and 0.2% CHAPS. In this assay, σ1R ligands dissolved in aqueous solutions display calcium- and concentration-dependent activity, altering the σ1R–TRP associations. If an organic solvent was required to incorporate the drug under study, such as dimethyl sulfoxide (DMSO) for pregnenolone sulfate, the DMSO had to remain below 1% in the assay buffer. Agarose pellets containing the bound proteins were obtained by centrifugation, and they were washed thrice in the presence of 3 mM CaCl_2_ and then solubilized in 2× Laemmli buffer, analyzing the σ1R/HINT1/CaM content in Western blots. The compounds studied were as follows: progesterone (#P7556, Sigma-Aldrich, Spain), pregnenolone sulfate (#P162, Sigma-Aldrich), S1RA (#16279, Cayman Chemical, USA), and PRE084 (#0589, Tocris Bioscience, UK) (see **Supplementary Figure 2**).

The σ1R/HINT1/CaM bound to the Sepharose-TRP sequences were resolved with Sodium dodecyl sulfate–polyacrylamide gel electrophoresis (SDS-PAGE) in 4–12% Bis–Tris gels (#NP0341, Invitrogen, Fisher Scientific, Spain), with 2-(N-morpholino)ethanesulfonic acid SDS (ME SDS) as the running buffer (#NP0002, Invitrogen). The proteins were transferred onto 0.2-μm Polyvinylidene difluoride (PVDF) membranes (#162-0176; BioRad) and probed overnight at 6°C with primary antibodies diluted in Tris-buffered saline (pH 7.7) (TBS) + 0.05% Tween 20 (TTBS): anti-σ1R (#42-3300, Invitrogen), anti-CaM (#05-173, Merck-Millipore), or the anti-HINT1 antibody produced in rabbits against the peptide sequence GYRMVVNEGADGGG (aa 93–106: Immunostep, Spain). All primary antibodies were detected using the appropriate horseradish-peroxidase-conjugated secondary antibodies. Thus, the blot areas containing the corresponding sizes of the cloned target proteins were selected for image capture and analysis. The Western blot images were visualized by chemiluminescence (#170-5061; BioRad) and recorded on an ImageQuant™ LAS 500 (GE). For each blot, the area containing the target cloned protein was typically selected. The device automatically captures the selected area and the associated software automatically calculated the optimal exposure time to provide the strongest possible signal from which the rest of the signals could be accurately quantified. For each group of immunosignals derived from the same cloned protein, the area of the strongest signal was used to determine the average optical density of the pixels within the object area/mm^2^ of all the signals (AlphaEase FC software). The gray values of the means were then normalized within the 8 bit/256 gray levels [(256 − computed value)/computed value].

### Statistical Analyses

The signals from the Western blot were expressed as the change relative to the controls, which were assigned an arbitrary value of 1. Statistical analyses were performed using the Sigmaplot/SigmaStat v.14 package [statistical package for the social sciences (SPSS) Science Software, Erkrath, Germany], and the level of significance was considered as *p* < 0.05. The data were analyzed using one-way ANOVA followed by Dunnett multiple comparisons against the control group.

## Results

### Interactions Between Sigma 1 Receptor, Calmodulin, and Histidine Triad Nucleotide-Binding Protein 1 with the N- and C-terminal Cytosolic Domains of Transient Receptor Potential Ankyrin Member 1/Melastatin Member 8/Vanilloid Member 1

**Figure 1:**
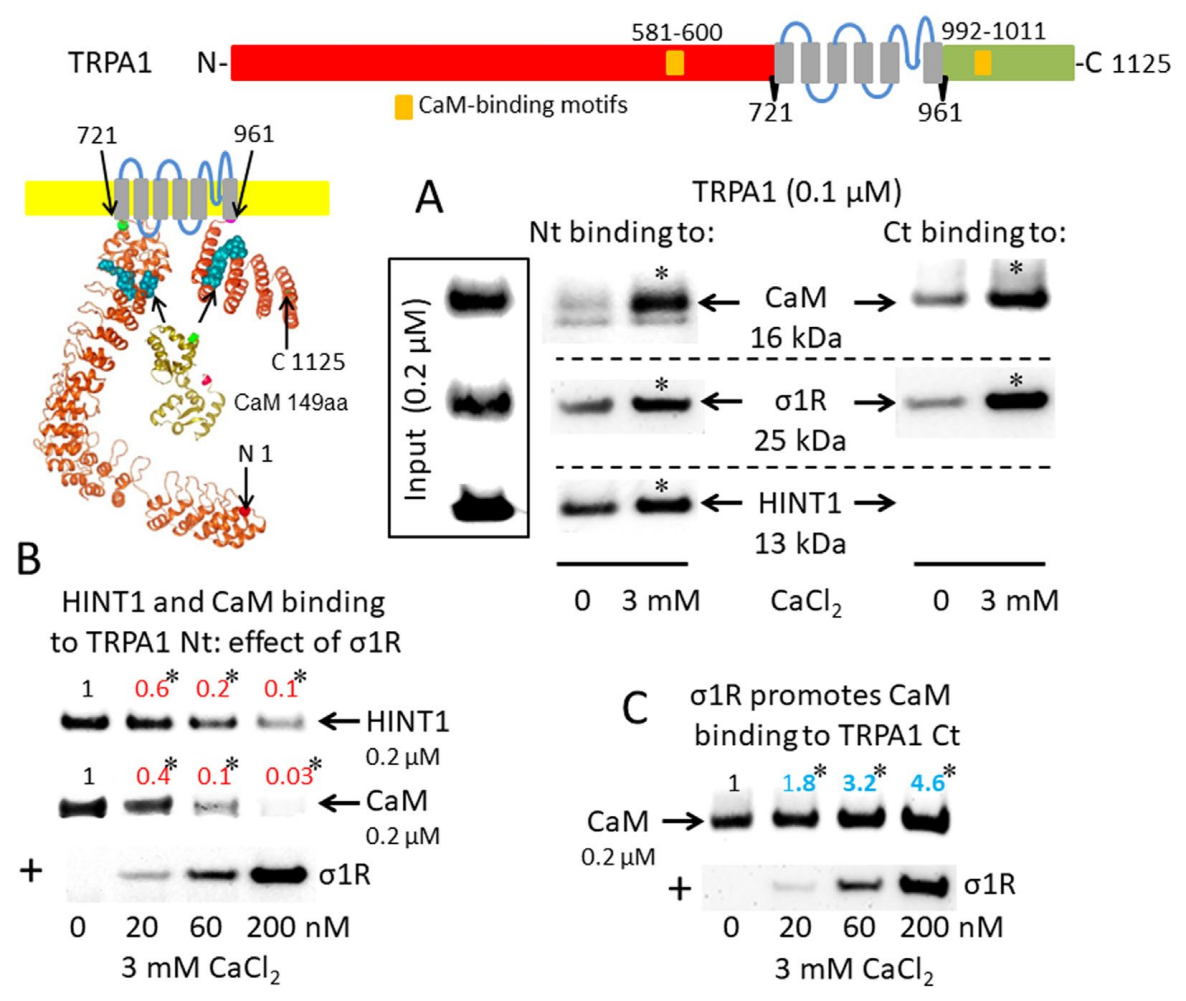
Sigma 1 receptor (σ1R), histidine triad nucleotide-binding protein 1 (HINT1), and calmodulin (CaM) binding to the transient receptor potential ankyrin member 1 (TRPA1) calcium channel. The TRP structural models shown were predicted by Novafold (DNASTAR Inc., Madison, WI, USA). Linear model: the N- and C-terminal cytosolic sequences are red and green, respectively, and the six transmembrane domains are in gray. Ribbon model: The 3D structure of N- and C-terminal sequences is shown; the CaM-binding motifs are indicated by blue spheres. **(A)** The *in vitro* interactions of the σ1R, CaM, and HINT1 with TRPA1 were evaluated in co-precipitation assays. Recombinant N- and C-terminal regions of TRPA1 (100 nM) were co-incubated in the presence and absence of 3 mM CaCl_2_, with the input of 200 nM CaM, σ1R, and HINT1. The TRPA1 N-terminus (aa 1–721) or the TRPA1 C-terminus (aa 961–1125) were immobilized by covalent attachment to NHS-activated Sepharose. Prey proteins alone did not bind to the blocked NHS-Sepharose (negative control). **(B** and **C)** Competition assays between the σ1R and CaM or HINT1 for binding to the N- and C-terminal regions of TRPA1. After incubation in 3 mM CaCl_2_, the TRPA1-bound proteins were detached and resolved by SDS-PAGE chromatography, and analyzed in Western blots. The assays were repeated at least twice, producing comparable results. For the interactions with increased concentrations of the σ1R (up to 200 nM), the data are shown relative to that obtained in the absence of the σ1R, with the control group arbitrary assigned a value of 1. *Significant differences with respect to the control group, ANOVA and Dunnett multiple comparisons vs. control group, *p* < 0.05. Representative blots are shown.

The activation of CaM by calcium provides a mechanism to rapidly regulate different signaling pathways and protein activities, such TRP cationic permeation. Protein analysis (DNASTAR NovaFold v15, Madison, USA) suggests the presence of CaM-binding motifs in the cytosolic sequences of the studied TRPs (Yap et al., 2000), and indeed, we identified a stable calcium-dependent interaction between CaM and the N- and C-terminal regions of TRPA1 (**Figure 1A**). The σ1R exhibited binding to both cytosolic regions of the TRPA1 channel, which increased considerably in the presence of 3 mM CaCl_2_. In contrast, the HINT1 protein interacted with the N-terminal but not the C-terminal domain of TRPA1. The formation of HINT1-TRPA1 complexes was moderately dependent on the calcium concentration (**Figure 1A**); importantly, the binding of the σ1R to the TRPA1 N-terminal domain prevented the binding of the HINT1 protein (**Figure 1B**). Although σ1R and CaM bind to the N- and C-terminal cytosolic regions of TRPA1, σ1R and CaM only compete for binding to the TRPA1 N-terminal domain (**Figure 1B**), whereas σ1R substantially enhanced the binding of CaM to the TRPA1 C-terminus (**Figure 1C**). Thus, σ1R competes with CaM and HINT1 for binding to the N-terminal domain of TRPA1.

**Figure 2:**
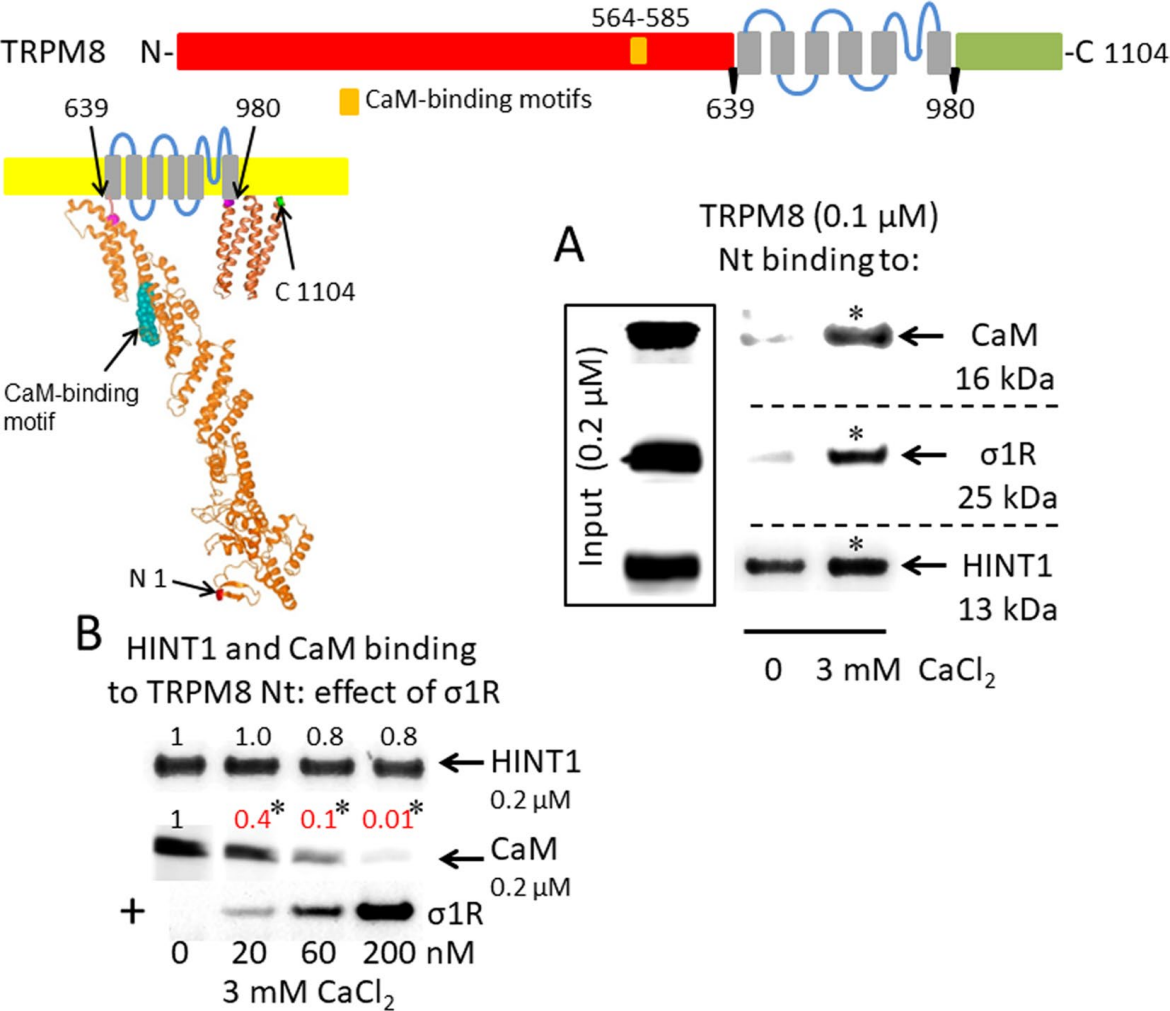
σ1R, HINT1, and CaM binding to the transient receptor potential melastatin member 8 (TRPM8) calcium channel. **(A)** Interactions between the σ1R, CaM, and HINT1 with the TRPM8 N-terminus (aa 1–639). Recombinant TRPM8 N-terminus (100 nM) was incubated with CaM, σ1R, and HINT1 (200 nM) in the presence or absence of 3 mM CaCl_2_. **(B)** Competition experiments to evaluate the interference of σ1R binding with that of CaM and HINT1 to the TRPM8 N-terminus (details as in **Figure 1**).

**Figure 3:**
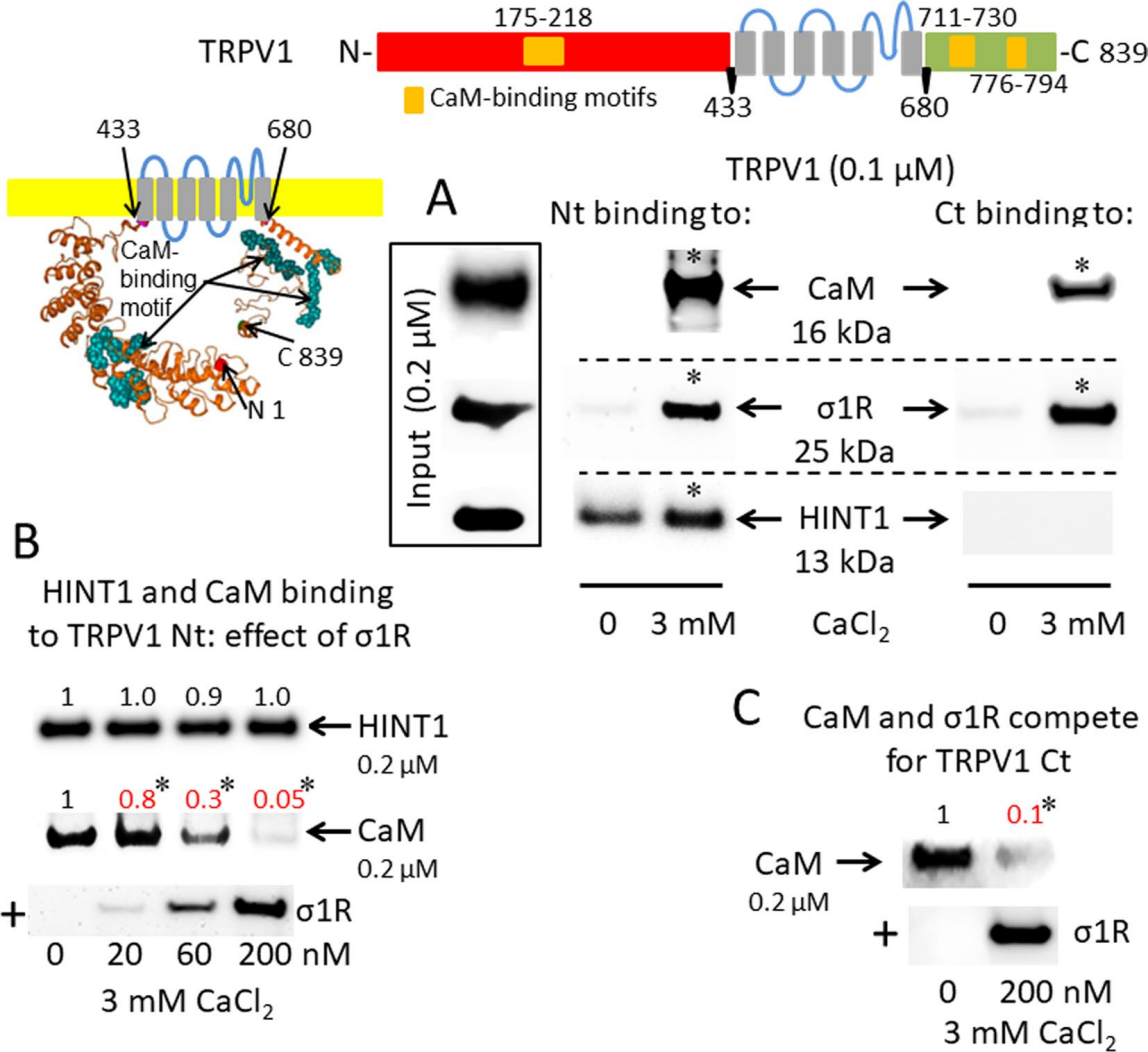
σ1R, HINT1, and CaM binding to the transient receptor potential vanilloid member 1 (TRPV1) calcium channel. **(A)** Interactions between σ1R, CaM, and HINT1 with the TRPV1. The recombinant TRPV1 N-terminus (aa 1–433) and C-terminus (aa 680–839) (100 nM) were incubated with CaM, σ1R, and HINT1 (200 nM) in the presence or absence of 3 mM CaCl_2_. **(B** and **C)** Competition assays between the σ1R and CaM or HINT1 for their binding to the N- and C-terminal regions of TRPV1 (details as in **Figure 1**).

A putative CaM-binding site was predicted in the TRPM8 N-terminal but not the C-terminal domain, and this binding was confirmed in our *in vitro* assays with the cloned proteins. Indeed, we detected the calcium-dependent binding of CaM, σ1R, and HINT1 to the N-terminus of TRPM8 (**Figure 2A**). In the absence of calcium, HINT1 interacted with the channel; the σ1R and CaM were virtually undetectable. The σ1R competed with CaM, but not with HINT1, for binding to the N-terminal region of TRPM8 (**Figure 2B**). Regarding the TRPV1 channel, its N-terminal ankyrin repeat domain and short distal C-terminal segment contained putative CaM-binding motifs. The σ1R, CaM, and, to a lesser extent, HINT1 all interacted with TRPV1, and their binding increased in the presence of 3 mM CaCl_2_ (**Figure 3A**). Similar to TRPA1, HINT1 bound to the N-terminal domain of TRPV1, but not to its C-terminus, although it did not apparently affect the binding of σ1R to this N-terminal region of TRPV1 (**Figure 3B**). However, the binding of σ1R hindered the interaction between CaM and the TRPV1 N- and C-terminal sequences (**Figure 3B and C**).

### Ligands of Sigma 1 Receptor Modify the Formation of the Sigma 1 Receptor– Transient Receptor Potential Ankyrin Member 1/Melastatin Member 8/Vanilloid Member 1 Complexes

**Figure 4:**
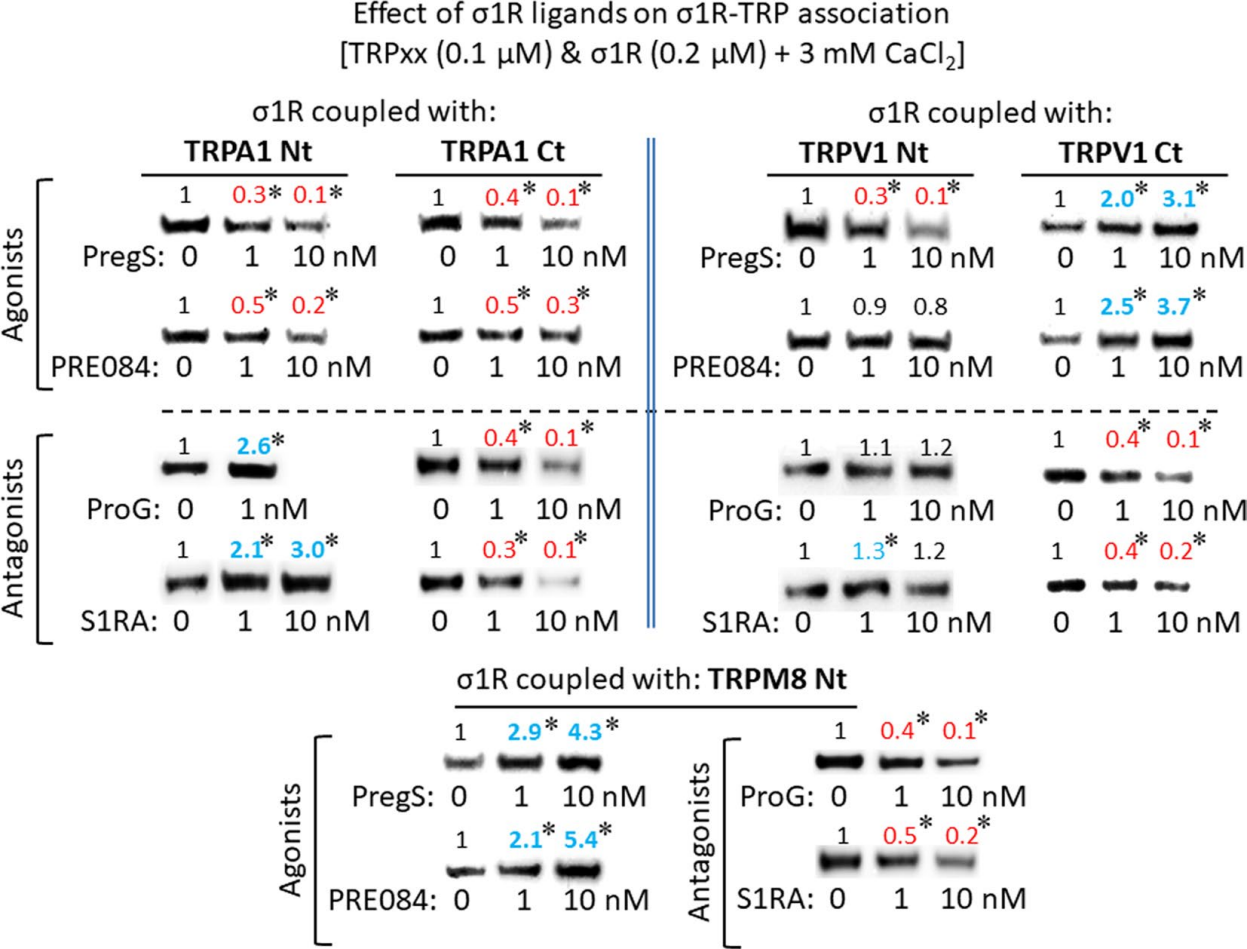
The effect of σ1R ligands on the σ1R–TRP interactions. Agarose-TRP was incubated with the σ1R and the agarose–TRP–σ1R complexes were separated from the free σ1R through three cycles of washing/resuspension. The agarose–TRP–σ1R complexes were then incubated for 30 min with rotation at room temperature (RT) in the presence of increasing concentrations of the σ1R ligands in a final volume of 300 µL (50 mM Tris–HCl [pH 7.5], 3 mM CaCl_2_, and 0.2% CHAPS). Finally, the σ1Rs that remained attached to the TRP were resolved by SDS-PAGE and evaluated in immunoblots. Agonists dampened on the association of the σ1Rs with TRPA1, while antagonist induced different effects on σ1Rs binding to the TRPA1 N- or C-terminal domains. The σ1R agonists produced different effects on the TRPV1-σ1R associations, and while the antagonists did not alter these associations at TRPV1 N-terminus, they did diminish these complexes at the TRPV1 C-terminal domain. The association of TRPM8 with the σ1R was enhanced by agonists and dampened by antagonists. The assays were performed twice, and each point was duplicated. Representative blots are shown. PregS, pregnenolone sulfate; ProG, progesterone. Details as in **Figure 1**.

**Figure 5:**
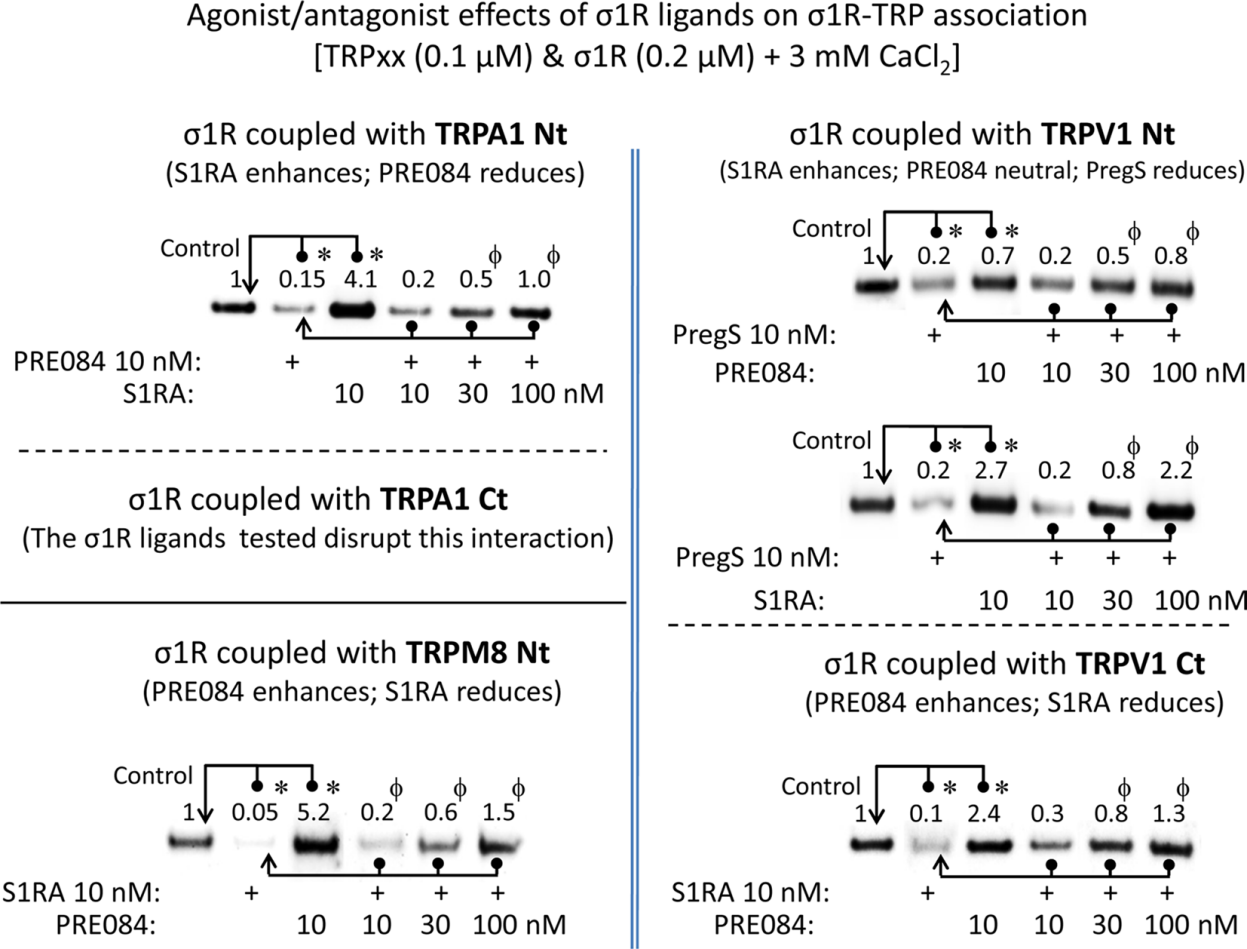
The effect of agonism/antagonism of σ1R ligands on σ1R–TRP interactions. Agarose TRP–σ1R complexes were incubated in the presence of the competing σ1R ligands as indicated. The σ1Rs that remained attached to the TRP were evaluated in immunoblots: *Significant differences with respect to the control group; ^ϕ^significant difference with respect to the group receiving only the ligand that diminished the σ1R–TRP interaction. ANOVA and Dunnett multiple comparisons vs. control group, *p *< 0.05. Details as in **Figures 1** and **4**.

For comparison with other reports, we will refer to the σ1R ligands as agonists and antagonists, based on their effects on the analgesic assays with morphine in rodents (Mei and Pasternak, 2002). In the presence of 3 mM CaCl_2_, the agonist pregnenolone sulfate blocked the interactions of the σ1R with both the N- and C-terminal domains of TRPA1 (**Figure 4**). Conversely, the antagonist progesterone enhanced the interaction of the σ1R with the N-terminal domain of TRPA1, while reducing its binding to the TRPA1 C-terminus. Pregnenolone sulfate also reduced the binding of the σ1R to the TRPV1 N-terminus, while substantially increasing the association of the σ1R with TRPV1 C-terminus and TRPM8 N-terminal sequence. Progesterone slightly augmented the interaction of σ1R with the TRPV1 N-terminal domain, while reducing σ1R binding to the TRPV1 C-terminus and TRPM8 N-terminus. The effects of neurosteroids on the interactions of σ1R with the three TRPs were mostly reproduced by exogenous ligands of this receptor. Thus, the selective antagonist S1RA modulated these associations to a similar extent as pregnenolone, and with the exception of TRPV1 N-terminus, the agonist PRE084 also reproduced the effects of pregnenolone sulfate (**Figure 4**). These data are summarized in **Table 1**. Conversely, the ligands that promote or did not alter the association of σ1R with TRP domains reduced the disruptive effects of other ligands on these σ1R–TRP complexes. For example, the interaction between pregnenolone sulfate and PRE084 at the TRPV1 N-terminus is of particular interest. Both ligands are considered agonists but pregnenolone sulfate weakened the σ1R–TRPV1 interaction at the N-terminus more effectively than PRE084. Hence, PRE084 diminished the capacity of pregnenolone sulfate to disrupt this particular σ1R interaction (**Figure 5**).

**Table 1:** Effect of sigma 1 receptor (σ1R) ligands on the association of σ1Rs with different signaling proteins.

Ligands	TRPA1 Nt	TRPA1 Ct	TRPV1 Nt	TRPV1 Ct	TRPM8 Nt	NR1 C0-C1	BiP
PregS	↓	↓	↓	↑	↑	↑ (a)	↓ (b)
PRE084	↓	↓	= *	↑	↑	↑ (a)	↓ (b)
ProG	↑	↓	=	↓	↓	↓ (a)	↑ (b)
S1RA	↑	↓	↑	↓	↓	↓ (a)	–

The arrows ↑ and ↓ indicate the enhancement or reduction in *σ*1R-target protein associations, while = denotes no change in such associations, *even though the ligand does not alter the *σ*1R–TRP interaction, the disruptive effect of pregnenolone sulfate (PregS) was impaired. References reporting the effects of *σ*1R ligands on the *σ*1R–NR1 or *σ*1R–binding immunoglobulin protein (BiP) interactions: (a) Rodríguez-Muñoz et al., 2015b, Antioxid. Redox Signal. 22: 799–818; (b) Hayashi and Su, 2007, Cell 131: 596–610.

PregS, pregnenolone sulfate; ProG, progesterone; BiP, binding immunoglobulin protein.

## Discussion

This molecular *in vitro* study demonstrates the physical interactions of σ1R with N- and C-terminal domains of the TRPA1, TRPM8, and TRPV1 calcium channels, and the dependence of its binding on calcium levels. Notably, calcium regulates σ1R binding to TRPs and also its interactions with the BiP protein in the ER (Hayashi and Su, 2007) and the NR1 C1 subunit of the NMDAR (Rodríguez-Muñoz et al., 2015a; Rodríguez-Muñoz et al., 2015b). Increases in calcium levels always promote the σ1R interaction with third partner signaling proteins, while calcium depletion reduces these associations. In the case of σ1R, a ligand-operated chaperone, depending on the interacting protein, BiP or NR1 C1, the same σ1R ligand either promotes the disruption of the complex or prevents the disrupting activities of other ligands. In this context, the present study confirmed the disparate activities of σ1R ligands to regulate the interactions of this chaperone with the cytosolic domains of the TRPA1, TRPM8, and TRPV1 channels. Based on all these observations, calcium emerges as the main known physiological regulator of σ1R chaperone activity. In this context, the regulation of σ1R interactions by endogenous molecules, such as steroids, *N*,*N*,-dimethyltryptamine, sphingosine, monoglycosylated ceramide, etc. (Hayashi, 2015), as well as exogenous compounds has attracted increasing pharmacological interest.

As described for the NMDAR (Ehlers et al., 1996), the calcium-activated CaM also reduces calcium permeation through TRP channels (Numazaki et al., 2003; Rosenbaum et al., 2004; Sarria et al., 2011; Hasan et al., 2017). The computer-predicted CaM-binding cytosolic regions in TRPA1, TRPM8, and TRPV1 coincided with the sites previously reported through mutation and sequence deletion assays (Numazaki et al., 2003; Rosenbaum et al., 2004; Hasan et al., 2017). The CaM binding motifs in NR1 C1 subunits overlap with the binding sites of the σ1R (Rodríguez-Muñoz et al., 2015b). Our observations also suggest that a similar phenomenon occurs in the TRPV1 N- and C-terminal regions and TRPA1 and TRPM8 N-termini. The TRPA1 C-terminus exhibit noticeable CaM binding, even in the absence of calcium, and this CaM binding motif must be located close to the σ1R binding site; thus, the chaperone positively influences CaM binding, suggesting a dual regulatory role for CaM in the function of this TRP. Indeed, at low calcium levels, CaM binds to TRPA1 C-terminus and increases calcium permeation through the channel; however, when calcium concentrations increase over a certain level, CaM, probably by binding to the N-terminus, desensitizes the TRPA1 channel (Hasan et al., 2017). Similar to the σ1R, ATP/Phosphatidylinositol 4,5-bisphosphate (PIP2) also prevents the desensitizing effect of CaM binding to the TRPV1 channel (Lishko et al., 2007); however, biochemical data addressing the possible competence of their binding to the receptor are unavailable. Regarding the physiological relevance of the present study, the σ1R always prevented CaM binding to the TRPs at matched concentrations, with the exception of the TRPA1 C-terminus, where σ1R binding cooperated with CaM binding. Since the σ1R negatively regulates the inhibitory effect of CaM on NMDAR function (Rodríguez-Muñoz et al., 2015b), a similar mechanism may regulate TRP activity. The binding of the σ1R to TRPs may favor the open probability of the channel, while CaM will reduce TRP activity by competing and diminishing σ1R binding. Hence, the resulting activity of the TRP calcium channels may depend on the concentrations of CaM and σ1R in their cytosolic environment.

The physiological mechanism regulating the NMDAR may be altered by exogenous compounds with antagonist activity at the σ1R, which promote CaM binding to the NR1 C1 subunit by disrupting the σ1R–NR1 C1 association, thereby inhibiting calcium permeation through the NMDAR. With respect to TRPs, the *in vivo* administration of the σ1R antagonists BD1063 or progesterone to mice promotes TRPV1 downregulation in DRG sensory neurons (Ortiz-Renteria et al., 2018), suggesting a protective effect of the σ1R chaperone on TRPV1 integrity. This pharmacological intervention may have disturbed the equilibrium between σ1R and CaM binding to the TRPV1, thus promoting an anomalous CaM-mediated inhibition of TRP function and the removal of the calcium channel from the neural membrane. The present molecular study showed how CaM, σ1R and HINT1 bind to cytosolic regions of the selected TRPs; however, the structural organization of the TRP channels raises a series of questions about the manner in which calcium-activated CaM regulates their function. TRPs are homotetramers and, in general, their N- and C-terminal domains contain CaM binding-motifs. Therefore, further studies are needed to elucidate whether CaM binding to just one site suffices to inhibit the channel or whether the extent of inhibition depends on the number of sites to which CaM binds in the channel, as suggested in a previous study (Rosenbaum et al., 2004). Another relevant issue is whether CaM binding to N-terminal regions collaborates with CaM binding to C-terminal regions, or if it accomplishes a different purpose. Further functional studies are required to address these questions.

The HINT1 and σ1R proteins are widely distributed in different tissues and are present in most cellular compartments (Hayashi and Su, 2007; Liu et al., 2008). At the neural membrane, HINT1 forms complexes with the cytosolic domains of different GPCRs, including MOR and cannabinoid type 1 (Guang et al., 2004; Sánchez-Blázquez et al., 2014). The σ1R also interacts with GPCRs and is implicated in the regulation of MOR activity (Sánchez-Blázquez et al., 2012; Rodríguez-Muñoz et al., 2015a). The MOR-associated HINT1 protein binds to the N-terminal domains of the TRPs evaluated in the present study, and this binding moderately increased in the presence of calcium. While the HINT1–NMDAR interaction is disrupted by σ1R binding, the HINT1–TRPM8/V1 N-terminal interactions were not affected by σ1R, which only impaired HINT1 binding to the TRPA1 N-terminal domain. These observations are compatible with MOR signaling, and probably that of other GPCRs, to influence TRP activity. Because HINT1 proteins interact with signaling proteins in zinc and redox-dependent and independent manners (Ajit et al., 2007; Rodríguez-Muñoz and Garzón, 2013), HINT1–TRP interactions may connect these channels to different signaling pathways in the membrane. These interactions may also influence events in the nucleus, where HINT1 exerts its anti-tumor activity and interacts with transcription factors (Weiske and Huber, 2005; Scholer et al., 2015).

The issue of ligand activity in modulating the binding of σ1Rs to different proteins is of particular pharmacological interest. In systems other than the regulation of MOR-mediated analgesia, σ1R pharmacology is complex, with exogenous ligands producing different effects depending on the system under study (Maurice and Su, 2009). Indeed, researchers have not clearly determined whether ligands are agonists or antagonists when they promote certain σ1R-mediated effects, such as neuroprotection or anti-convulsing effects (Rodríguez-Muñoz et al., 2018; Sánchez-Blázquez et al., 2018). Thus, the modulatory effects of σ1R ligands on the interactions of this chaperone are dissimilar and, for new σ1R interactors, unpredictable. As aforementioned, this characteristic was initially observed for σ1R interactions with BiP and NR1 C1 subunits of the NMDAR, where the effects of the ligands tested were completely opposite. The agonists disrupt σ1R–BiP complexes and antagonists prevent the effect of agonists, but the antagonists disrupt σ1R–NR1 C1 complexes and agonists oppose the effects of the former ligands. The associations of σ1Rs with cytosolic regions of TRPA1, TRPM8, and TRPV1 did not escape this complex regulation by σ1R ligands, and thus the effects of agonists and antagonists on these complexes did not show a common pattern but rather varied, depending on the channel and even the cytosolic region considered. The data from the literature and the present study suggest the existence of at least three main types of interactions of the σ1R with other proteins. The first type accounts for the negative regulation of MOR analgesia by σ1Rs, in which the neural glutamate NMDAR plays an essential role (Garzón et al., 2012; Rodríguez-Muñoz et al., 2015b). Antagonists disrupt and agonists promote σ1R binding to the TRPM8 N-terminus and TRPV1 C-terminus, where σ1R and CaM compete for binding to the TRP channel. The second classification involves the TRPA1 N-terminal region and probably the TRPV1 N-terminus, and it corresponds to the interaction of the σ1R with BiP in the ER (Hayashi and Su, 2007). In this situation, agonists disrupt σ1R binding and antagonists promote or fail to modify it, although they block the effects of agonists. Again, the σ1R and CaM compete for binding to the TRP channel. In the third category, agonists and antagonists disrupt σ1R binding to the TRPA1 C-terminal domain.

Overall, the effects of different ligands on the interactions of this chaperone with its targets are similar to those described for the agonists of most GPCRs, which is actually known as agonist bias. This phenomenon is typical of exogenous ligands, although some reports have described this signaling pathway preference for GPCRs with various endogenous ligands, e.g., the endogenous opioids and the MOR (Thompson et al., 2015). The cytosolic regions of a given 7-TM GPCR bind to different G proteins and certain ligands exhibit a preference to activate the receptor when it is coupled to some but not all the regulated G proteins. We have characterized this situation for clonidine and agonists of the MOR and delta-opioid receptors (Sánchez-Blázquez et al., 1999; Sánchez-Blázquez et al., 2001); the affinity exhibited by opioid agonists, but not antagonists, depends on the class of G protein coupled to the MOR (Garzón et al., 1998). More relevantly, those opioid agonists exhibiting biased activity through discrete combinations of MOR with G proteins bind to the other combinations, but without triggering the signaling pathways. Thus, agonist-biased MOR opioids also display antagonism toward the effects of other biased or unbiased agonists while acting on MOR-G protein combinations on which the former are inactive (Sánchez-Blázquez and Garzón, 1988; Garzón et al., 1994). Thus, the GPCR field and concrete studies of the MOR have shown that ligands may behave as biased agonists and even antagonists, depending in the class of G protein coupled to the opioid receptor.

The situation described for GPCR and G proteins compares satisfactorily with the findings being documented for the σ1R in its interactions with signaling proteins. The G proteins that interact with the GPCR determine the agonist/antagonist activity of the ligands, and the different signaling proteins that associate with the σ1R determine the activity of a given ligand, namely, dissociation or stabilization. Thus, the σ1R would compare with a GPCR, as the interacting proteins, BiP, NR1 C1, and TRP cytosolic domains, play similar roles to the different classes of G proteins, Gi, Go, Gz, Gq, etc. As observed for the interactions of the MOR with different G proteins (Garzón et al., 1998), the conformation adopted by σ1R when it binds to the NR1 C1 and BiP must differ, and the conformation when binding to the TRPA1 C-terminus may also be different. The association of the σ1R with the TRPV1 N-terminus was diminished by pregnenolone sulfate but only mildly by PRE084. In this situation, the latter ligand binds to the σ1R and diminishes the effects of the neurosteroid. Antagonism was also detected, and the σ1R ligands that did not influence σ1R–TRP interactions diminished the effects of the active ligands. Thus, one should not expect any particular σ1R ligand to exert a similar effect on all TRP channels, as its activity is likely to depend on the channel type and even on the particular cytosolic region analyzed.

TRP channels have been implicated in a wide range of physiological activities. In peripheral nerves, ganglia, and the substantia gelatinosa of the dorsal horn, the activation of TRP channels by different agents contributes to pain perception and even allodynia. The expression of certain TRPs in supraspinal areas, such as those included in this study, suggest that they may participate in other signaling processes that are yet to be defined. The precise role of HINT1 in modulating TRPM8 and TRPV1 channels remains to be explored, although the proposed connections between TRPV1 and MOR (Ortiz-Renteria et al., 2018) suggest that the σ1R–HINT1 protein complex functions to connect calcium channels such as the TRPs and NMDAR with this GPCR. Thus, σ1R ligands exhibit biased activity to regulate subsets of σ1R interactions with third partner proteins, and this finding may be exploited in the development of site-specific drugs with therapeutic significance.

## Author Contributions

JG and PS-B designed the research, wrote the manuscript, and obtained the funding. EC-M and YO performed the experiments and the statistical analysis of data. MM helped to perform the analysis with constructive discussions. All authors approved the final manuscript.

## Funding

This work was supported by MICINN Plan Nacional I+D+i [grant number RT 2018-093677-B-100]. EC-M was supported by a Grant from MECD [FPU 15/02356].

## Conflict of Interest Statement

MM is employed by Company Esteve Pharmaceuticals.

The remaining authors declare that all the research presented here was conducted in the absence of any commercial or financial relationships that could be construed as a potential conflict of interest.
