# Supplementary material for: Ligands Exert Biased Activity to Regulate Sigma 1 Receptor Interactions With Cationic TRPA1, TRPV1, and TRPM8 Channels
Source: Front Pharmacol. 2019 Jun 12;10:634. doi: 10.3389/fphar.2019.00634 (PMC6582314; doi:10.3389/fphar.2019.00634)
Supplement: Supplementary file 1 [file Table_1.docx]

**Supplementary Fig. 1.**

**The absence of an interaction between GST and the σ1R**. Recombinant σ1R (200 nM) was incubated for 30 min at room temperature (mixed by rotation) with GST and GST-TRPA1 Ct in 300 μL of a buffer containing 50 mM Tris-HCl [pH 7.5] and 0.2% CHAPS, in the presence of 2.5 mM CaCl_2_. Subsequently, 40 μL of glutathione sepharose (GE Healthcare, GE#17-0756-01) was added to the mixture, which was then recovered by centrifugation, washed three times, solubilized in 2x Laemmli buffer and analyzed in Western blots.


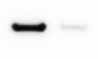


**WB: σ1R**

bound to GST

bound to GST-TRPA1 Ct

-TRP

-TRP

-TRP

-TRP

-TRP

-TRP

-TRP

-TRP

-TRP

-TRP

-TRP

-TRP

-TRP

-TRP

-TRP

-TRP

Prot. A

Prot. A

Prot. A

Prot. A

Prot. A

Protein A

Protein A

Protein A

Protein A

Protein A

Protein A

Agarose beads; TRP Nt or Ct covalently attached.

Target Proteins A & B: σ1R, CaM or HINT1

SDS-PAGE

Protein B

Protein A

-TRP

-TRP

-TRP

-TRP

-TRP

-TRP

-TRP

-TRP

-TRP

-TRP

-TRP

-TRP

-TRP

-TRP

-TRP

-TRP

Prot. B

Prot. A

Prot. A

Prot. A

Prot. B

Protein A

Protein B

Protein A

Protein A

Protein B

Protein A

Protein A

Protein B

Prot. B

**Supplementary Figure 2**.

**Typical pull-down assays**. The Nt or Ct regions of TRPA1, TRPV1 and TRPM8 Nt were covalently attached to agarose beads (see methods) and incubated with free σ1R, CaM or HINT1 (left panel), or with combinations of two of these free proteins (right panel). The assays were conducted in the presence of absence of calcium. The agarose protein complexes were recovered and washed to remove the unbound proteins, and analyzed in Western blots. Antibody binding to the cloned proteins (σ1R, CaM or HINT1) was visualized by chemiluminescence and recorded using an ImageQuant™ LAS 500 (GE). The protein standards (STD) are colored, and the capture device combines the chemiluminescence data and STD images. Because the assays performed with recombinant proteins identified proteins of the expected sizes and the antibodies can only label proteins of that size, the capture of the data was optimized. Thus, the area containing the recombinant protein was typically selected in each blot. The device automatically captures the area selected and it calculates the optimal exposure time to provide the strongest possible signal to achieve an accurate comparison between the samples. For quantification, protein immunolabeling was measured using the area of the strongest signal of each studied group of samples studied (AlphaEase FC software).

Typical TRP-single protein assays:


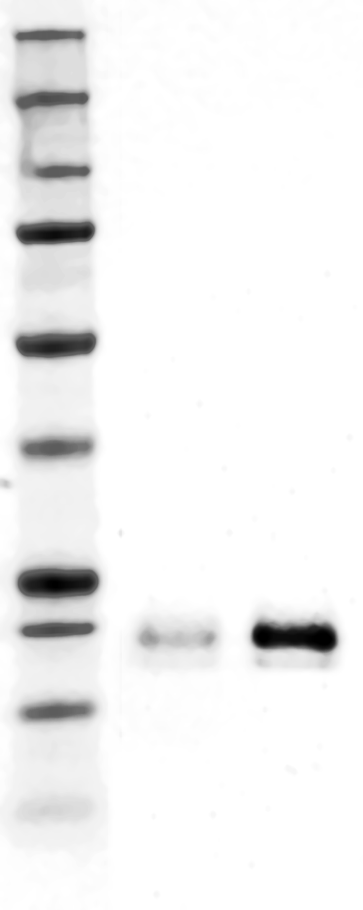


**TRPA1 Nt** & **CaM**

0 3 mM

CaCl_2_

STDs

kDa

50

37

25

20

15

10

75

100

150

250

STDs

kDa

50

37

25

20

15

10

75

100

150

250

**TRPV1 Ct** & **σ1R**


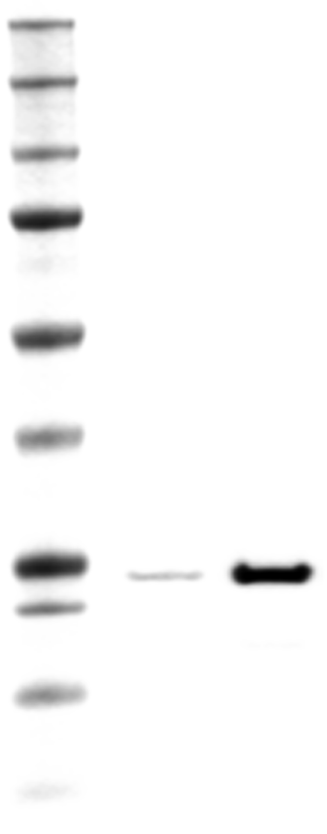


0 3 mM

CaCl_2_

STDs

kDa

50

37

25

20

15

10

75


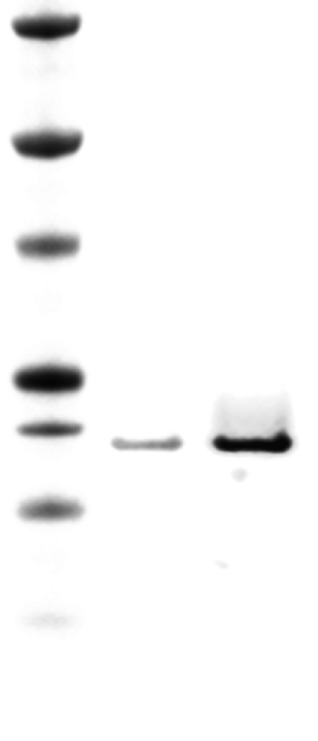


**TRPA1 Ct** & **CaM**

0 3 mM

CaCl_2_

**TRPM8 Nt** & **HINT1**


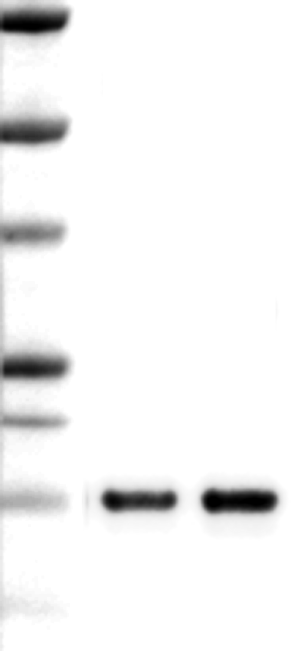


STDs

kDa

50

37

25

20

15

10

75

0 3 mM

CaCl_2_

**CaM**

**CaM**

**σ1R**

**HINT1**

Typical TRP-double protein assay. Sequential immunodetection of σ1R and CaM:

**σ1R & CaM exhibit competitive binding to TRPA1 Nt**


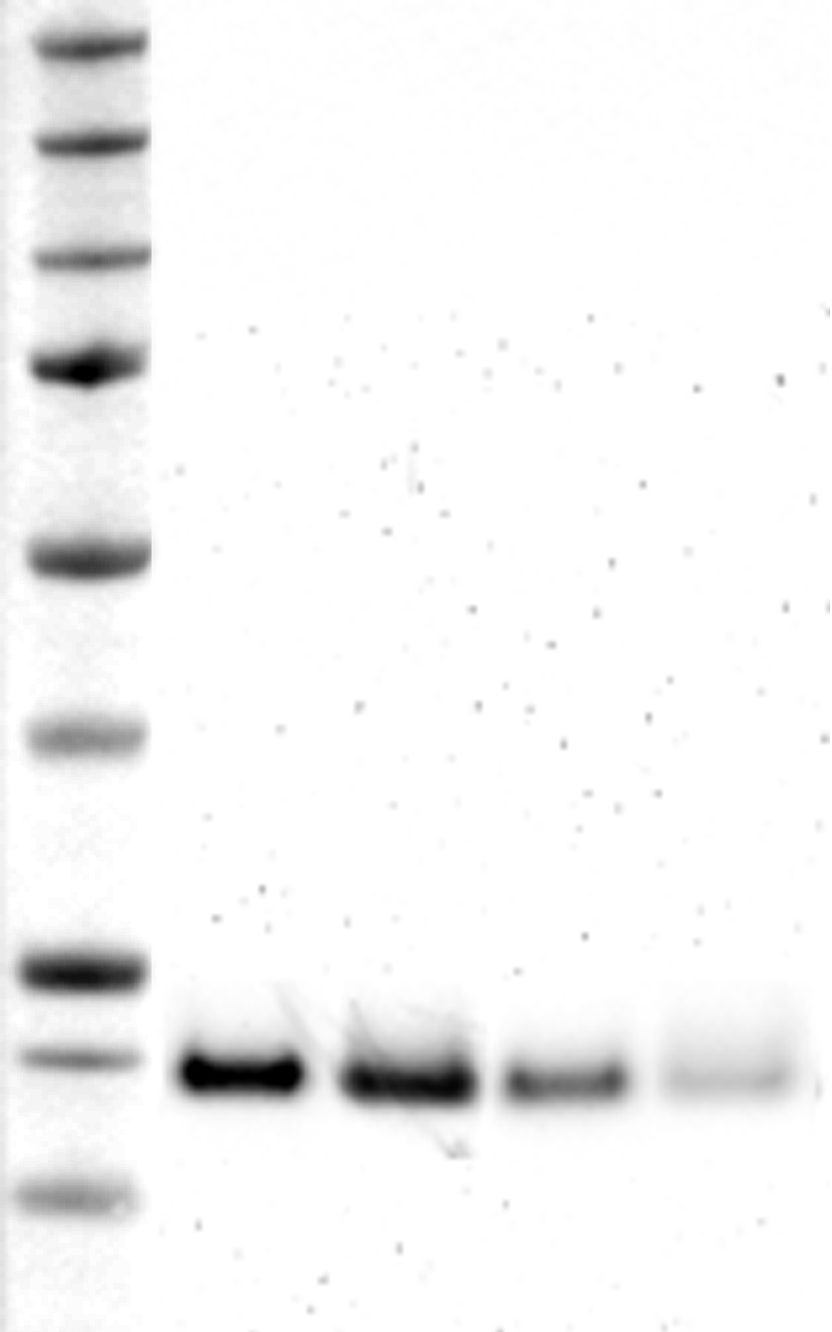


**WB: CaM**

**CaM**


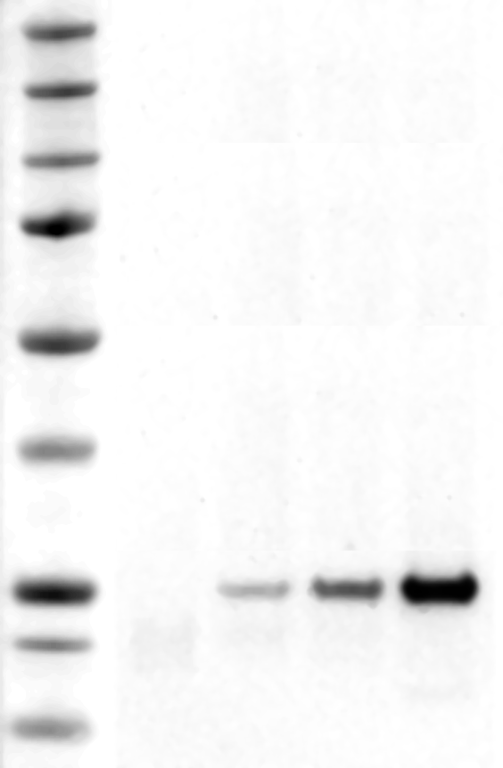


0 20 60 200 nM

σ1R

**WB: σ1R**

STDs

kDa

50

37

25

20

15

75

100

150

250

**σ1R**

0 20 60 200 nM

σ1R
